# The Role of Shame, Stigma, and Family Communication Patterns in the Decision to Disclose STIs to Parents in Order to Seek Support

**DOI:** 10.3390/ijerph20064742

**Published:** 2023-03-08

**Authors:** Emily Scheinfeld

**Affiliations:** School of Communication & Media, Kennesaw State University, Kennesaw, GA 30144, USA; escheinf@kennesaw.edu; Tel.: +1-470-578-2572

**Keywords:** parent–child relationship, communication, health disclosure decision-making model, emerging adults, sexually transmitted infections (STIs), disclosure

## Abstract

Emerging adulthood is identified as a time of personal growth wherein emerging adults engage in sexual exploration and risky behaviors, potentially resulting in the contraction of a sexually transmitted infection (STI). Due to the continued reliance on parents for support during this developmental period, emerging adults (EAs) may need to disclose their STI status to their parents. This study applies the health disclosure decision-making model (DD-MM) to extend our understanding of EA disclosures of sensitive health information such as STIs to parents. Data were collected from 204 college students. The results of mediational analyses provided some support for the mediating effects of family communication patterns on the relationship between relational quality and illness assessment (i.e., stigma) and willingness to disclose in a given scenario. The theoretical and practical implications of this are discussed.

## 1. Introduction

Sexually transmitted infections (STI) remain a public health concern, with over 26 million new cases a year arising [1]. About half of STI cases are documented to be among adolescents and emerging adults (EAs) (ages 18–26) [2] due to reasons that include behaviors unique to the demographic. As EAs embark on the transition into adulthood, they engage in personal and sexual exploration [3] to form their own attitudes, beliefs, and behaviors [4]. EAs are therefore more likely to partake in risky sexual behavior and have multiple and simultaneous sexual partners [5]. Unfortunately, they are also less likely to use protection [6].

STIs have consequences for the infected individual’s psychological wellbeing [7], self-esteem, sexual self-concept, [8], and physical health [9]. Disclosing an STI status may be important to coping with the diagnosis by receiving support in the process. However, many infected individuals avoid disclosing their status due to the potential risk of rejection by a loved one, as well as feelings of anxiety, stress, and shame [8]. For EAs, the tension associated with disclosing an STI status is amplified. Past research has demonstrated that the felt shame and stigma could prevent EAs from disclosing their STI status to parents and casual sexual partners [10]. Parents may serve as a safe source to disclose and receive support necessary to cope with a new STI status. However, the desire to divulge to parents is hindered by a want and need to conceal personal private information in order to retain autonomy [3] and preserve the parent–child relationship, which may be challenged with the revelation of a child’s sexual activity.

We apply the health disclosure decision-making model (DD-MM) [11] to the parent–EA relationship in the context of STIs because it provides a framework to examine and understand the variables that influence EAs’ decision to disclose to their parents. Research has examined disclosing an STI to partners [7,8] and to children (e.g., [12]); however, there is little to no research conducted specifically surrounding the disclosure to a parent, even in hypothetical disclosures or likely decisions to disclose. Communication between parents and children about an STI diagnosis is important to investigate due to parents’ ability to provide substantial support for an illness during the emerging adulthood years.

## 2. Literature Review

### 2.1. The Transition to Emerging Adulthood

The years between 18 and 26 years of age are often identified as the transition from adolescence to adulthood. EAs embark on a period of personal growth and sexual exploration [3], and form their own attitudes, behaviors, and beliefs [4]. The ramifications of the ever-lingering hookup culture, accidentally catching feelings [13], and online dating and social networking sites [14] leave EAs with some uncertainty, intense emotions, the need to address their own emotions, and the need to gain some awareness. However, during this process of individuation [15,16], adult children may desire more restrictive boundaries around information and between themselves and their parents, especially as it pertains to risky and sexual behaviors [3]. Continued reliance on parental figures, however, makes privacy boundaries between a parent and child blurred and difficult to navigate [17], yielding a level of relational uncertainty [18]. This uncertainty and felt tension surrounding the disclosure of personal information to parents is often amplified in difficult contexts, rife with anxiety and stress [19], and by the discomfort associated with talk about sex with parental figures [20].

Sex communication with parents has been widely explored, including talk about sex [21] and its impact on [22] safe sex practices and sexual health. This research has been invaluable to STI/HIV prevention interventions [23,24], which have reversely been imperative in promoting parent–child communication about sex [25]. These conversations about sex, sexual health, and sexual identity, while important, remain generally uncomfortable and difficult for most families, who have often relied on outdated scripts discussing what most children and young adults can find online [26], rather than the topics found to be most useful [27]. Consequently, disclosing an STI is also difficult, and may subject an infected individual to moral condemnation, shame, stigma, and the association of the infected individual with disreputable groups, in the eyes of their family member [28], that is, tribal associations come with the stigma of having a chronic STI, putting people who contract them among those who are dirty, irresponsible, promiscuous, and other “undesirable” groups (e.g., men who sleep with men (MSM), sex workers, and drug users) [28]. Past research has revealed that disclosing an STI to a parent does carry shame and stigma with it [10]. However, divulging a status to parents is also coupled with the revelation of past sexual activity, which involved persons may have strategically avoided during a child’s transition into adulthood [3,29]. This revelation could shift the nature of the parent–child relationship by having to accept the child’s role as an adult, as well as impact the degree of trust, intimacy, and communication within the relationship [8]. Yet, to receive social support, disclosure is necessary.

### 2.2. The Disclosure Decision-Making Model and Decisions to Disclose

The DD-MM [11] integrates and provides “a framework to predict decisions to disclose… [including the elements of] disclosure uncertainty, managing uncertainty regarding the information, the relationship, and efficacy in evaluating whether or not to share.” The model has been used for a variety of disclosures, including transgender identity [30,31], various health issues (e.g., epilepsy [32] and mental health [33,34]), and even within a variety of contexts (e.g., the workplace [35] and social networking sites [36]). The model has remained informative to help researchers understand how individuals process the decision to disclose these various pieces of private information to specific others. For our purposes, the DD-MM posits that infected individuals first assess the health information or diagnosis before disclosing [11,37,38]. Given the shame and stigma often associated with STIs [27], especially when thinking about disclosing to a parent [10], EAs may reconsider communicating about their diagnosis. If the “risk is not too great and the process lends itself to continued consideration of revealing, the discloser will then move to consider” the receiver of the disclosure [11]. For EAs, assessing a shameful and stigmatizing diagnosis such as an STI may increase the perceived risk associated with disclosing to a parent [10]. However, due to the need for support, EAs may disclose to a third party instead. Consistent with the health DD-MM, the following is posited:

**H1:**  *Negative assessment of STIs is (a) negatively associated with likelihood of disclosing to a parent and (b) positively associated with likelihood of disclosing to a third party.*

As part of the DD-MM, Greene [11,38] argues individuals assess the receiver based on the relational intimacy and quality with the confidant; thus, a positive assessment of the relationship with the confidant moves individuals to decide to disclose [11,38]. According to the DD-MM, better relational quality should outweigh the desire to manage a particular identity to parents. Specifically, love and trust may increase the likelihood of disclosing to a parent and decrease the likelihood of relying on and disclosing to a third party. Thus:

**H2:**  *Relational quality is (a) positively associated with likelihood of disclosing to a parent and (b) negatively associated with likelihood of disclosing to a third party.*

If, after assessing the information and the receiver, the benefits outweigh the risks associated with disclosing, infected individuals then assess their disclosure efficacy. Disclosure efficacy is defined as the ability and confidence to disclose health information [11]. Family communication patterns (FCP) [39] are a useful way to conceive of disclosure efficacy because family communication environments shape children’s social behaviors and beliefs [40]. Moreover, perceptions of the ability to disclose within a family relationship cannot be divorced from the family context; if EAs believe they can disclose to their parents, it is because patterns of communication within the family make it normative to do so.

Family communication patterns are comprised of two dimensions: conversation and conformity orientations [39]. Conversation orientation represents “the degree to which families create a climate in which all family members are encouraged to participate in unrestrained interactions about a wide array of topics” [39]. Highly conversation-oriented families feel free to interact, share ideas, and express concerns. While Bridge and Schrodt [41] found conversation orientation did not encourage EAs to share private information with parents, EAs’ need for tangible support within the context of an STI diagnosis may differentiate between personally private information and what is private yet shared within the family (i.e., family-private, e.g., [42]). Conformity orientation indicates “the degree to which family communication stresses a climate of homogeneity of attitudes, values, and beliefs” [39]. High conformity families focus on the family interest, and while these families can have positive relationships, EAs may be more likely to retain personally private information for the sake of homogeneity within the family [41].

**Figure 1:**
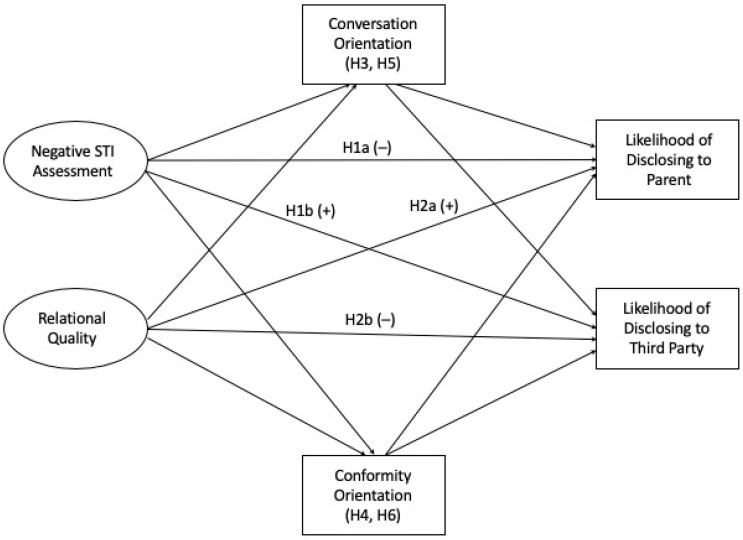
Hypothesized model.

Disclosing an STI to a parent is a difficult process, potentially requiring a great deal of efficacy. Families high in conversation talk about a variety of topics and allow members to express their concerns, so it is likely that individuals in this type of family also talk about health issues and behaviors, that is, the efficacy to communicate personal information such as an STI status may be a hallmark of individuals from conversation-oriented families, especially as EAs from high conversation families are more likely to disclose health issues to parents [43], partially due to increased communication efficacy [44], a consistently strong indicator of health self-disclosure [45]. On the other hand, EAs from more conformity-oriented families may feel less able to disclose their STI status. Specifically, EAs from families that value high conformity not only are less likely to disclose health issues, but also less likely to disclose health issues even if they also come from high conversation families, as the need to conform constrains any conversation [43]. Hays and her colleagues [43] do note that, no matter the family communication orientation an EA comes from, they are still less likely to disclose sensitive issues, as compared to non-sensitive issues, such as chronic pain. Therefore, this study further asserts the importance of looking at FCP as the variable of *disclosure efficacy* within DD-MM, as EAs may instead disclose to a third party such as a friend. The following hypotheses are posited (see Figure 1):

**H3:**  *Conversation orientation mediates the relationships between assessment of STIs and (a) the likelihood of disclosure to a parent and (b) the likelihood of disclosure to a third party.*

**H4:**  *Conformity orientation mediates the relationships between assessment of STIs and (a) the likelihood of disclosure to a parent and (b) the likelihood of disclosure to a third party.*

**H5:**  *Conversation orientation mediates the relationships between relational quality and (a) the likelihood of disclosure to a parent and (b) the likelihood of disclosure to a third party.*

**H6:**  *Conformity orientation mediates the relationships between relation quality and (a) the likelihood of disclosure to a parent and (b) the likelihood of disclosure to a third party.*

## 3. Materials and Methods

### 3.1. Participants

The participants included 204 students, aged 18–25 (*M* = 20.59; *SD* = 1.69), from a large Southwestern university who were recruited through announcements on a departmental website and in undergraduate communication courses. Most were female (*n* = 166; 86.5%) and most identified as non-Hispanic/White (*n* = 115; 59.9%), followed by Hispanic or Latino (*n* = 43; 22.4%). The analyses in this research were based on data from 192 participants, as 12 did not complete several measures.

### 3.2. Procedures

Recruitment emails and website posts included a link to an online survey. After consenting to participate, participants were asked to report on scales intended to measure trust, love for and liking of their parents, family communication patterns (FCP), and stigma and shame toward STIs. Participants were also presented with a variety of situations in which they were diagnosed with an STI and asked how likely they would be to tell their parent. They were also asked how likely they would disclose an STI to a third party. Demographic information was also collected.

### 3.3. Measures

#### 3.3.1. Information Assessment

Participants’ evaluations of health information were operationalized as shame and stigma. To assess shame, six items measured a sense of contamination and shame towards an STI [46]. Participants reported, on a 7-point Likert-type scale (1 = strongly disagree, 7 = strongly agree), the extent to which they endorsed statements such as, “Getting a sexually transmitted disease means a person is dirty.” Alpha reliability for the shame toward STIs scale was 0.93 (*M* = 2.67, *SD* = 1.40).

Stigma was measured through items assessing reactions to STIs and STI-related testing [46]. Using a 7-point Likert-type scale (1 = strongly disagree; 7 = strongly agree), participants responded to five items pertaining to stigma towards STIs (e.g., “Getting a sexually transmitted infection would make me feel lonely”). Reliability for the scale was 0.85 (*M* = 3.48, *SD* = 1.50).

#### 3.3.2. Relational Quality

Participants’ love for and liking of parents and parental trust were used to assess overall relational quality. Love and liking were assessed using a modified version of Rubin’s Loving and Liking scale [47]. The items were modified to address the relationship between parent and child by changing the prompt from “my partner” to “my parent.” Participants used a Likert-type scale (1 = not true; 9 = definitely true) to indicate their response to items addressing feelings of love and liking towards their parent. Sample loving items included “I feel I can confide in my parent about virtually anything”. Liking items included “My parent is the sort of person whom I myself would like to be.” The reliability of the loving scale was 0.84 (*M* = 5.12, *SD* = 1.03), and was 0.91 for the liking scale (*M* = 5.58, *SD* = 1.02).

Parental trust was assessed by eight items using a 7-point Likert-type scale (1 = strongly disagree; 7 = strongly agree) to assess trust in their parental figures. This measure is a modified version of the Dyadic Trust Scale [48], where “my partner” was changed to “my parent”, for example, “I feel that I can’t trust my parent completely” and “My parent is perfectly honest and truthful with me.” Similar modifications have been made in the past [49]. The reliability of the parental trust scale was 0.87 (*M* = 5.54, *SD* = 1.12).

#### 3.3.3. Disclosure Efficacy

For disclosure efficacy, we used the child version of the Revised Family Communication Patterns measure (RFCP) [39]. Using a Likert-type scale (1 = strongly disagree; 7 = strongly agree), participants responded to 15 items about conversation orientation and 11 items addressing conformity. Example items included “My parents often ask my opinion when the family is talking about something” (conversation) and “When I am home, I am expected to obey my parents’ rules” (conformity). Individual scales were created for conversation orientation (α = 0.94; *M* = 4.95; *SD* = 1.21) and conformity orientation (α = 0.86; *M* = 3.68; *SD* = 1.12).

#### 3.3.4. Likelihood of Disclosing to Parent

Participants were asked to report the likelihood they would disclose an STI status to their parent (1 = extremely unlikely, 7 = extremely likely) based on six items created for this study. Each item began with, “How likely are you to tell your parent you have contracted an STI if you…”. Items included, “…if you have your own health insurance,” “…if you are covered under your parents’ insurance”, “…if the STI is chronic”, “…if the STI is acute”, “…if the STI conditions require medical treatment” and “…if the STI conditions do not require medical treatment”. An exploratory factor analysis using principal axis-factoring extraction methods and varimax rotation showed all six items loaded onto one factor that accounted for 74.42% of the variance in the factor. We retained all six items (α = 0.93; *M* = 3.68; *SD* = 1.12).

#### 3.3.5. Likelihood of Disclosing to Third Party

Participants were asked to rate “How likely are you to tell someone else (e.g., your best friend or confidant) that you were diagnosed with an STI before you told your parent?” on a Likert-type scale (1 = extremely unlikely, 7 = extremely likely). The mean of the item was 5.22 (*SD* = 1.94).

## 4. Results

### 4.1. Analyses

Descriptive analyses were conducted to examine the mean, standard deviation, and distribution of all primary variables. Correlations between all variables were also examined as preliminary analyses before testing any research questions. The majority of the variables were relatively normal in nature.

We used structural equation modeling to assess Hypotheses 1–6. We used a covariance matrix as the input to AMOS 25.0 and estimated the parameters using maximum likelihood procedures. We chose guidelines for fit indices a priori: the model’s chi-square should not be significant, the model’s comparative fit index (CFI) should exceed 0.95, and the standardized root mean square residual (SRMR) and the root mean square error of approximation (RMSEA) should not exceed 0.08. To estimate the indirect effects, as well as their significance in both the simple and multiple mediation models, we used 95% bias-corrected bootstrap confidence intervals (CIs) with 5000 samples [50]. Measurement models were constructed to ensure that stigma and shame did comprise the negative assessments of STIs, and that love, liking, and parental trust contributed to the relational quality latent factor. Both measurement models had zero degrees of freedom and thus had a “perfect fit”. Importantly, each of these loaded sufficiently onto their respective latent factors (shame = 0.87; stigma = 0.81; loving = 0.75; liking = 0.89; and parental trust = 0.84).

**Figure 2:**
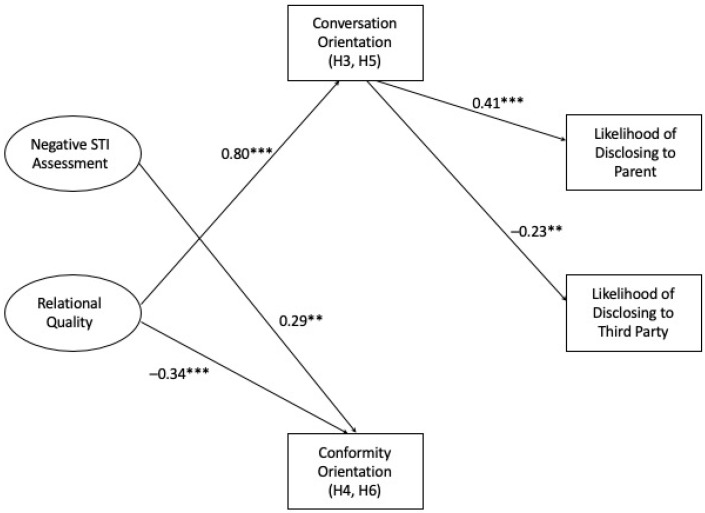
Final model with standardized paths. Note: ** *p* < 0.01; *** *p* < 0.001.

The initial model showed a good fit of the data: χ^2^ = 19.15, *df* = 19, *p* = 0.45, CFI = 1.00, RMSEA = 0.01, SRMR = 0.03. However, there were several insignificant paths, which we trimmed using empirical procedures outlined by Kline beginning with the least significant path [51]. The final model (Figure 2) also showed the good fit of the data: χ^2^ = 29.92, *df* = 26, *p* = 0.27, CFI = 0.99, RMSEA = 0.03, SRMR = 0.05. In addition, a chi-square difference test between the initial model and the final model suggests that deleting the insignificant paths did not worsen the fit of the model: χ^2^ = 10.77, *df* = 7, *p* = 0.15.

### 4.2. Hypothesis 1

Hypothesis 1 predicted that negative assessments of STIs would be negatively associated with the likelihood of disclosing to a parent and positively associated with the likelihood of disclosing to a third party. This hypothesis was not supported, as the total and direct effects of STI assessment on these variables could not be tested (i.e., insignificant paths). Thus, H1 was not supported.

### 4.3. Hypothesis 2

Hypothesis 2 predicted that relational quality would be positively associated with the likelihood of disclosing to a parent and negatively associated with the likelihood of disclosing to a third party. Similarly, because the paths between relational quality and the likelihood of disclosure to a parent and a third party were insignificant, direct effects could not be tested. Thus, H2 was not supported.

### 4.4. Hypothesis 3 and Hypothesis 5

Though these direct effects were not significant, mediation may still exist [50]. Hypothesis 3 predicted that conversation orientation mediated the relationships between STI assessment and (a) the likelihood of disclosure to a parent and (b) the likelihood of disclosure to a third party. Hypothesis 5 predicted that conversation orientation mediated the relationships between relational quality and (a) the likelihood of disclosure to a parent and (b) the likelihood of disclosure to a third party. As seen in Figure 2, relational quality was significantly related to conversation orientation. In turn, conversation orientation was positively related to the likelihood of telling a parent but negatively related to the likelihood of telling a third party. Specifically, the standardized total effect (including the total indirect effect, because the direct effect alone was not significant) of relational quality on the likelihood of disclosing to a parent was 0.32, *p* < 0.001, 95% CIs [0.22, 0.42]. The standardized total effect (including the total indirect effect, because the direct effect alone was not significant) of relational quality on the likelihood of disclosing to a third party was −0.19, *p* < 0.01, 95% CIs [−0.29, −0.07]. The model accounted for 16% of the variance in the likelihood of disclosing to a parent and 5% of the variance in the likelihood of disclosing to a third party. Thus, H3 and H5 were supported.

### 4.5. Hypothesis 4 and Hypothesis 6

Turning to conformity orientation as mediator, Hypothesis 4 predicted that conformity orientation mediates the relationships between assessment of STIs and (a) the likelihood of disclosure to a parent and (b) the likelihood of disclosure to a third party. Hypothesis 6 predicted that conformity orientation mediates the relationships between relation quality and (a) the likelihood of disclosure to a parent and (b) the likelihood of disclosure to a third party. Conformity was not related to the likelihood of disclosing to either a parent of a third party. Thus, H4 and H6 were not supported.

## 5. Discussion

The purpose of this study was to apply the DD-MM to emerging adults’ disclosures of their STI status to their parent. In doing so, we wanted to test whether assessments of the relationship and health information (i.e., shame and stigma) influence perceptions of disclosure efficacy (i.e., conversation or conformity family communication patterns), and in turn, if efficacy predicts the likelihood of disclosure.

Perhaps the most central finding of the study is that conversation orientation fully mediated the relationship between EAs’ evaluations of their relationship with their parent and their likely decisions to disclose an STI to that parent, despite the sensitive nature of the disclosure, which had often restricted the disclosure in past studies [43]. Close and satisfying relationships with parents promote more open and frequent communication that makes sensitive and personal disclosures, such as the diagnosis of an STI, more acceptable and welcomed. Of the goals that EAs have for disclosing an STI status to a parent, requesting support is likely to be a prominent one [4]. Therefore, by disclosing an STI status to a parent, these EAs may be more likely to receive the financial, informational, and emotional support from parents that cover doctor appointments, and treatments, as well as creating a safe environment for EAs to efficaciously cope with emotional outcomes that stem from contracting STIs (e.g., decrease in self-esteem, desirability, and issues affecting future relationships) [8].

Conformity, however, was not a significant mediator of the relationship between relational quality and information assessment and the disclosure of an STI. Individuals in this study from more conformity-oriented families view STIs as more stigmatizing and shameful and have lower quality relationships with their parents, that is, being a part of a family that emphasizes shared beliefs, morals, behaviors, and so forth, is able to constrain even high-communication families and negate the assessment of the receiver of the information (relational quality with parents). However, conformity orientation was not related to the likelihood of disclosing to a parent or to a third party. This is an important implication of our findings—who do EAs turn to if not their parent or a close friend? EAs may feel as if they have to manage their STI independently, which could make it more difficult for them to garner the support (instrumental, emotional, or otherwise) needed to manage their STI. These individuals may rely more heavily on their partner, thus diminishing the need to disclose to a parent or to a third party. This study indicated the third party individual would likely be a “best friend or confidant”; thus, participants may also have interpreted that as a romantic partner, if they had one. In addition to the “likelihood of disclosure to a third party”, future research should consider conversations about or the disclosure of the STI status to the partner that potentially gave the EA the STI—given the hookup culture EAs are often a part of—as well as the current sexual or romantic partner, if they are different individuals.

### 5.1. The Role of Stigma and Shame

Of the many factors comprising EAs’ assessments of an STI diagnosis (prognosis, relevance to others, and symptoms), this study focused on their perceptions of stigma and shame. Negative assessment of STIs was not associated with the likelihood to disclose an STI to parents or a third party, nor was it related to conversation orientation, findings which are different to that of past studies [43], potentially due to the introduction of the other variables as part of DD-MM. Moreover, while there was variance in the stigma measure (*M* = 3.48; *SD* = 1.50), there was a slight floor effect, that is, most of the participants did not experience much stigma towards STIs. This may be due to a variety of factors, including increased sex education; better understanding of the infections; and the ability to better treat many STIs, thus allowing the infection to subside. There is also increased knowledge that infections such as HPV infect over half of sexually active individuals in the United States at some point in their life and can disappear without treatment. Such prevalence may decrease the stigma surrounding STIs, impacting its role in how sensitive it is of a health issue to some EAs.

The culture surrounding sex during emerging adulthood may have also decreased the role of shame and stigma. EAs are exploratory in nature when it comes to risky behaviors [3], increasingly engaging in the hookup culture and sexual behaviors outside of committed relationships [52]. This behavior is common and widely accepted [52]. However, participation in the hookup culture compounds the risk of infection, since those who do so are more likely to have multiple and simultaneous sexual partners [5] and are less likely to use condoms [6]. A lack of concern toward contracting an STI and the prevalence of STIs may very well be responsible for the lack of reported stigma and shame in this study. Future research should examine other possible constructs involved in assessing STI diagnoses and the health information surrounding them as EAs decide to disclose (or not) these to parents.

### 5.2. Limitations and Future Directions

Although this study aims to make unique contributions to communication and health scholarship, it does have limitations. First, this study relied on an undergraduate student research pool that was relatively homogeneous, with most participants identifying as White (non-Hispanic; *n* = 60.5%) and female (*n* = 86.5%). A more diverse sample would allow for a more inclusive understanding of the communicative processes surrounding STI disclosure to one’s parents that may significantly differ due to possible differences in culture surrounding sex based on geographical location, family values, ethnicity, or religiosity. Additionally, several of the measures were developed for the purpose of this study. More research is needed to test the validity and reliability of these measures. Future research should also delve deeper into both the significant and insignificant results of this study to pinpoint what does work, that is, what variables are most important in EAs disclosing to parents when it comes to sensitive health issues, such as STIs, that need treatment and social support to improve the ability to cope with the diagnosis, as well as better health communication in the future (e.g., safe sex conversations with romantic partners).

### 5.3. Practical Implications

The health DD-MM was developed to understand the various processes involved in disclosing health information within interpersonal contexts [11]. The results of this study provide an opportunity to develop stronger theory-based health interventions targeting sexual behavior in EAs. With the understanding gained from the results of this study, including regarding the role of family communication in diagnosis disclosure decisions, the shame and stigma surrounding STIs in the eyes of EAs, and to whom EAs may turn after the decision process happens, interventions may be able to assist in guiding parent–child conversations about STIs, that is, interventions may be better armed to encourage the updating of scripts for sex communication, to cover a wider array of topics, and to encourage conversation with their child earlier on. Alongside other research on effective intervention campaigns [25], families may be better prepared for sex communication, and consequently may become the place to which children turn when in need later on in life. Interventions may also be developed to help guide EAs to ask for help once diagnosed. Disclosing to parents may not be easy, but it may provide EAs with the social support to reduce the psychological injury associated with contracting an STI. Having a positive disclosure experience within the parent–child dyad may also provide tangible support to control the infection, reducing the risk of infecting others, in addition to emotional support that allows infected individuals to better cope. In addition to being able to manage the health issue at hand, they may also adopt advantageous coping strategies and resilience [53], which can assist in rebuilding their relationships and sexual confidence [8]. As such, infected individuals may become more assertive when having to disclose sexual pasts with romantic partners, helping to reduce the spread of STIs. In addition to testing the model in its entirety, further research should examine this process of the feedback and reassessment component of the DD-MM.

## 6. Conclusions

The overarching goal of this study was to better understand EAs’ perceptions of their decisions about whether to disclose an STI diagnosis or status—a private and sensitive subject—with a parent. This study offers support for the influence that relational quality with a parent has on the likelihood that EAs will disclose an STI to a parent, or whether they will turn to a third party, instead. Additionally, this study highlights the important role of familial communication patterns in the decision-making process. Ultimately, the insight provided by this study may allow researchers to apply the health disclosure decision-making model more comprehensively and within the context of the family unit.

## Data Availability

The data presented in this study are available on request from the corresponding author. The data are not publicly available due to IRB protocol at the time of approval.
